# Interaction of electromagnetic radiation in the 20–200 GHz frequency range with arrays of carbon nanotubes with ferromagnetic nanoparticles

**DOI:** 10.3762/bjnano.6.106

**Published:** 2015-04-24

**Authors:** Agylych Atdayev, Alexander L Danilyuk, Serghej L Prischepa

**Affiliations:** 1 Belarusian State University of Informatics and Radioelectronics, P. Browka St. 6, 220013 Minsk, Belarus

**Keywords:** carbon nanotubes, electromagnetic radiation, ferromagnetic nanoparticles, magnetic dipole, magnetic nanocomposite, resonance circuit

## Abstract

The interaction of electromagnetic radiation with a magnetic nanocomposite based on carbon nanotubes (CNT) is considered within the model of distributed random nanoparticles with a core–shell morphology. The approach is based on a system composed of a CNT conducting resistive matrix, ferromagnetic inductive nanoparticles and the capacitive interface between the CNT matrix and the nanoparticles, which form resonance resistive–inductive–capacitive circuits. It is shown that the influence of the resonant circuits leads to the emergence of specific resonances, namely peaks and valleys in the frequency dependence of the permeability of the nanocomposite, and in the frequency dependence of the reflection and transmission of electromagnetic radiation.

## Introduction

Magnetic nanocomposites consisting of ferromagnetic nanoparticles embedded into a matrix material are currently the subject of intensive study. The properties of such materials can be tuned by the external magnetic field, spin-polarized current or electromagnetic radiation. In conventional ferromagnetic materials, the magnetic properties are determined by the domain structure and domain walls within the grains. In nanostructured materials, the magnetic properties and the static and dynamic magnetic behavior are controlled by the interparticle exchange interaction, which gives rise to a new generation of devices with improved characteristics and new functionalities. Such magnetic nanocomposites are prospects for memory storage, emission and high frequency devices.

New magnetic nanocomposites based on carbon nanotubes (CNTs) [1–3] are very promising for high frequency applications [4–14] such as transmission lines, mixtures and detectors [15–17], antennas and absorbing materials [18–26]. The absorption properties of CNT-based nanocomposites are primarily determined by the dielectric loss [27]. However, the intercalation of magnetic nanoparticles into the CNT matrix leads to the increase of the absorption properties due to the induced magnetic loss [24,28–31]. In order to predict the absorption properties of CNT-based nanocomposites, the realization of a wide variety of experimental investigations and the development of theoretical approaches, which take into account the different parameters of nanocomposites, are necessary.

In particular, the interaction of electromagnetic radiation (EMR) with CNT-based magnetic nanocomposites is developed in several directions [4–8]. One of the main problems is related to the elucidation of the absorption mechanisms of the EMR by such complex nanocomposites composed of a porous carbon matrix, ferromagnetic nanoparticles and the interfaces between them [8]. In such systems, accounting for the properties of the carbon matrix, nanoparticles and interfaces becomes of great importance [8]. This issue can be taken into account when considering the CNT-based nanocomposite as a system of such components comprised of a CNT conducting resistive matrix, ferromagnetic inductive nanoparticles and the capacitive interfaces between the CNT matrix and the nanoparticles. Therefore, the development of models to adequately describe the absorption properties of CNT-based magnetic nanocomposites in a wide frequency range is an important task.

Based on a previously developed approach [8], in this paper, the interaction of EMR with a CNT-based nanocomposite in the frequency range 20–200 GHz is theoretically studied. Special attention is given to the role of the resonance resistive–inductive–capacitive (*R_i_L_i_C_i_*) circuits, which leads to a deeper understanding of the problem. Such nanocomposites can be easily synthesized in situ during the CNT growth by chemical vapor deposition, which involves carbon decomposition of an organic precursor with 3*d* catalytic metals such as Fe, Ni and Co [32].

## Model

Before describing our theoretical approach in detail, we first specify the object of investigation targeted in this work. We consider the CNT-based nanocomposite synthesized by floating catalyst chemical vapor deposition (FCCVD) employing ferrocene Fe(C_5_H_5_)_2_ as the source of catalytic iron-based nanoparticles (NPs). Under standard FCCVD conditions (i.e., synthesis temperature: 1150 K, ferrocene concentration: 5–10 wt %, injection rate of the Ar carrier gas: 100 cm^3^/min, growth duration: 1 min), the multiwall CNTs are formed. The height of the structure is approximately 50 μm and the average CNT diameter is less than 40 nm [8]. The conductivity of such samples is usually in the range of 100–120 (Ω·m)^−1^ at room temperature [8] and at liquid helium temperature, this decreases to 40–50 (Ω·m)^−1^. The catalytic, single crystalline, iron-based NPs for the above mentioned ferrocene content are distributed both inside and outside the CNTs and are covered by a carbon shell which prevents their oxidation [8,32–33]. The average size of the NPs is slightly less than the CNT diameter, and lies in the range of *a* = 20–30 nm [34], and are therefore considered as a single domain [35]. The main phase of the NPs is iron carbide (Fe_3_C) with an orthorhombic crystalline structure, and their saturation magnetization at room temperature is *M*_sat_ ≈ 90 A·m^2^/kg and the Curie temperature was measured as *T*_C_ = 481 K [32]. Raman spectroscopy can usually reveal slight defects in such CNTs [8,33–34].

The model of the interaction of EMR with CNT-based nanocomposite used in this work is based on a previously developed approach [8]. This approach relies on the modified Bruggeman effective medium theory, which takes into account the conductive magnetic particles randomly distributed in the medium [36–37], and was developed to determine the reflection (*R*) and transmission (*T*) coefficients of the EMR for nanostructured magnetic composites at frequencies above 1 GHz. It takes into account both the magnetic properties of the NPs and the transport, structural and magnetic properties of the CNT matrix. The interface between the NPs and the CNT matrix was also considered and characterized by the wave impedance, *Z_i_*. The calculated *R* and *T* coefficients adequately describe the experimental data for the X and K_a_ bands [8].

Here, we modify this approach [8] by specifying the expressions for the permeability and permittivity of the nanocomposite taking into account the possible resistive, capacitive and inductive coupling between the components of the sample. Indeed, in the frequency range of tens or hundreds of GHz, the microwave properties of the nanocomposite should strongly depend not only on the magnetic and dielectric properties of the CNT matrix material and magnetic inclusions, but also on the contribution of the resistive–inductive–capacitive coupling (circuits) which arise in such a complex system. These couplings, which are due to the presence of eddy currents in the nanocomposite, could be described by *R_i_L_i_C_i_* contours. On the other hand, the CNT-based nanocomposite can be represented as a matrix of CNTs in which ferromagnetic NPs are randomly distributed [8]. Each NP is coated by a protective shell (interface) according to the experimental findings [32–33]. We suppose that the *R_i_L_i_C_i_* contours are formed primarily due to the resistance of the CNT matrix, the NP inductance, and the capacitance of the interfaces. For simplicity, we also assume that the *R_i_*, *L_i_*, and *C_i_* model parameters are constant for each of the cases considered in this article. Physically, this means that all the NPs are of the same size and all interfaces have the same capacitance. Under these assumptions, the *R_i_L_i_C_i_* circuit describing the nanocomposite properties is resonant, that is, it has its own resonance frequency.

To model the permeability of such a nanostructured composite, we obtained the following modified expression:

[1]
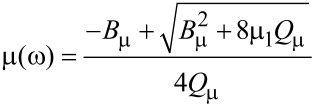


where

[2]
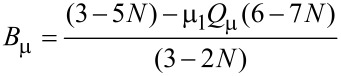


[3]
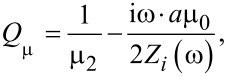


µ_1_ and µ_2_ are the relative permeabilities of the carbon matrix and the ferromagnetic NPs respectively, *a* is the NP diameter, ω is cyclic frequency of EMR, µ_0_ is magnetic constant, and *N* is the volume NP concentration.

For the permittivity, the following modified expression was deduced:

[4]
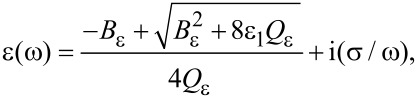


where

[5]
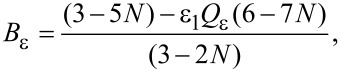


[6]
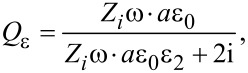


ε_1_ and ε_2_ are the relative permittivities of the carbon matrix and the NP, respectively, ε_0_ is permittivity of vacuum, and σ is the specific conductivity of CNT-based nanocomposite.

The reflection coefficient is determined as

[7]
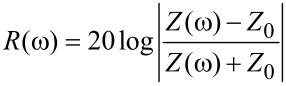


where 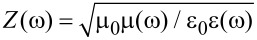, and *Z*_0_ = 377 Ω is the characteristic impedance of the plane wave in vacuum.

The transmission coefficient, which determines the efficiency of shielding, consists of the absorption, reflection, and multi-reflection processes,

[8]
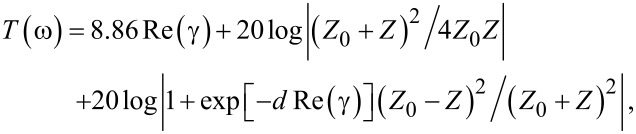


where *d* is the nanocomposite thickness, and


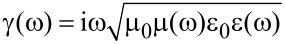


is the propagation coefficient.

The impedance of the nanocomposite depends on the contribution of the resonance circuits containing active resistance, *R_i_*, inductance, *L_i_*, and capacitance, *C_i_*. Their resonant frequency is 
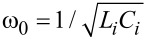
. We considered both the series and parallel connection of these elements that form the circuits, where the impedances are determined by known expressions.

For the series circuit, the impedance is

[9]
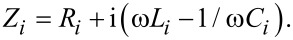


For the partially parallel, *R_i_L_i_C_i_* circuit (assuming the resistance *R_i_* is in series with the inductance *L_i_*), the impedance is

[10]
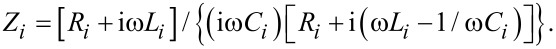


For the series–parallel circuit (*L_i_* and *C_i_* are in parallel and *R_i_* is in series with them), the impedance is expressed as

[11]
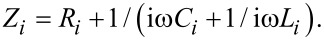


For a fully parallel circuit (*L_i_*, *C_i_* and *R_i_* are in parallel), the impedance is

[12]
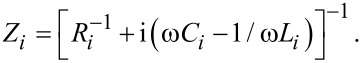


Equations 1, 4, 7 and 8, together with Equations 2, 3, 5, 6 and 9–12 were applied for the calculation of the frequency dependent ε(ω) [38], µ(ω), *R*(ω) and *T*(ω). For all results reported in this work, the specific conductivity was set as σ = 120 (Ω·m)^−1^ [8].

Other model parameters were similar to that reported in [8], but for the sake of convenience, we summarize them together in Table 1 with the parameters of the *R_i_L_i_C_i_* circuits used in the calculations. The dimensions and properties of the nanocomposite components provide a range of *L_i_* and *C_i_* parameters corresponding to the pH and pF, respectively, and *R_i_* values in the range of mΩ. This choice of circuit parameters matches with the specified frequency range of 20–200 GHz.

**Table 1:** Values of input parameters used in the model.

Parameter	Unit	Values			

*a*	nm	20–30			
*N*	a.u.	0.1 ± 0.01			
μ_1_	a.u.	(3 ± 0.5) + i(0.35 ± 0.1)			
μ_2_	a.u.	(7 ± 2) + i(0.5 ± 0.2)			
ε_1_	a.u.	(10 ± 0.5) + i(0.1 ± 0.05)			
ε_2_	a.u.	(2 ± 0.2) + i(0.1 ± 0.05)			
Re(ε*_a_*^a^)	a.u.	14 ± 2			

		Type of circuit			

		series	partially parallel	series–parallel	fully parallel

*R_i_*	Ω	0.003–0.02	0.01–0.02	0.005–0.01	0.002–0.01
*C_i_*	pF	2–20	1–15	1–15	1–10
*L_i_*	pH	0.2–2	0.01–0.1	0.01–0.12	0.01–0.15

^a^Permittivity of the nanocomposite.

## Results and Discussion

**Figure 1:**
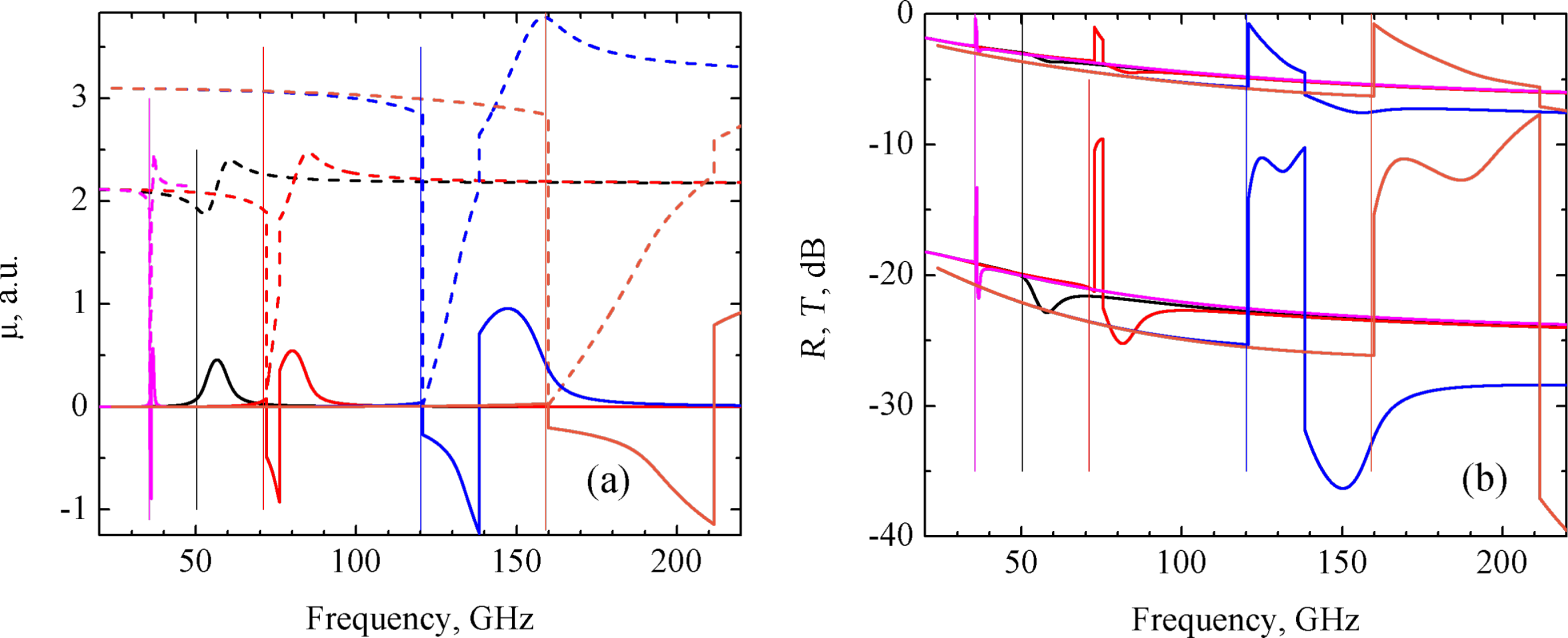
Frequency dependence of the real (dashed lines) and imaginary (solid lines) parts of the permeability (a), and of the reflection, *R* (upper curves), and transmission, *T* (lower curves), coefficients (b) for the CNT-based nanocomposite consisting of a series *R_i_L_i_C_i_* circuit. From left to right: *R_i_* = 0.015 Ω, *L_i_* = 2 pH, *C_i_* = 10 pF (ω_0_ = 35.6 GHz) (magenta lines); *R_i_* = 0.015 Ω, *L_i_* = 0.5 pH, *C_i_* = 20 pF (ω_0_ = 50.3 GHz) (black lines); *R_i_* = 0.015 Ω, *L_i_* = 0.5 pH, *C_i_* = 20 pF (ω_0_ = 71.2 GHz) (red lines); *R_i_* = 0.012 Ω, *L_i_* = 0.35 pH, *C_i_* = 5 pF (ω_0_ = 120.3 GHz) (blue lines); *R_i_* = 0.012 Ω, *L_i_* = 0.2 pH, *C_i_* = 5 pF (ω_0_ = 159.1 GHz) (orange lines). The resonance frequency of the *R_i_L_i_C_i_* circuit, ω_0_, is represented by a vertical line of corresponding color.

For the case of the series circuit, for which the impedance is expressed by Equation 9, the nonlinearity of µ(ω) close to the resonance frequency and, consequently, of *R*(ω) and *T*(ω) were obtained (Figure 1a,b, curves 1 and 2). In particular, at some values of the circuit parameters, a strong reduction of the µ(ω) dependence is observed, followed by smooth peak (Figure 1a). Correspondingly, the reflectivity and transmission first increase significantly and then drop (Figure 1b). Consequently, depending on the circuit parameters, one can observe either a gradual change in the resonance for reflection and transmission, or a sharp change of these coefficients. The second type of resonance is characterized by large steps in the *R* and *T* frequency dependence; namely, *R* increases almost to 0 dB, and *T* changes from −22 dB to −10 dB. The step width could reach 10–15 GHz.

It was found that the reduction of *L_i_* and *C_i_* results in a broadening and shift of the peaks towards higher frequencies. This result is depicted in Figure 1a,b. Note that the nanocomposite resonance always occurs at a frequency close to the resonance frequency of the circuit, ω_0_. The resonance frequency is depicted in Figure 1 by thin vertical lines. The increase in the inductance by several orders of magnitude results in the narrowing of the resonance and the reduction of the resonance frequency.

**Figure 2:**
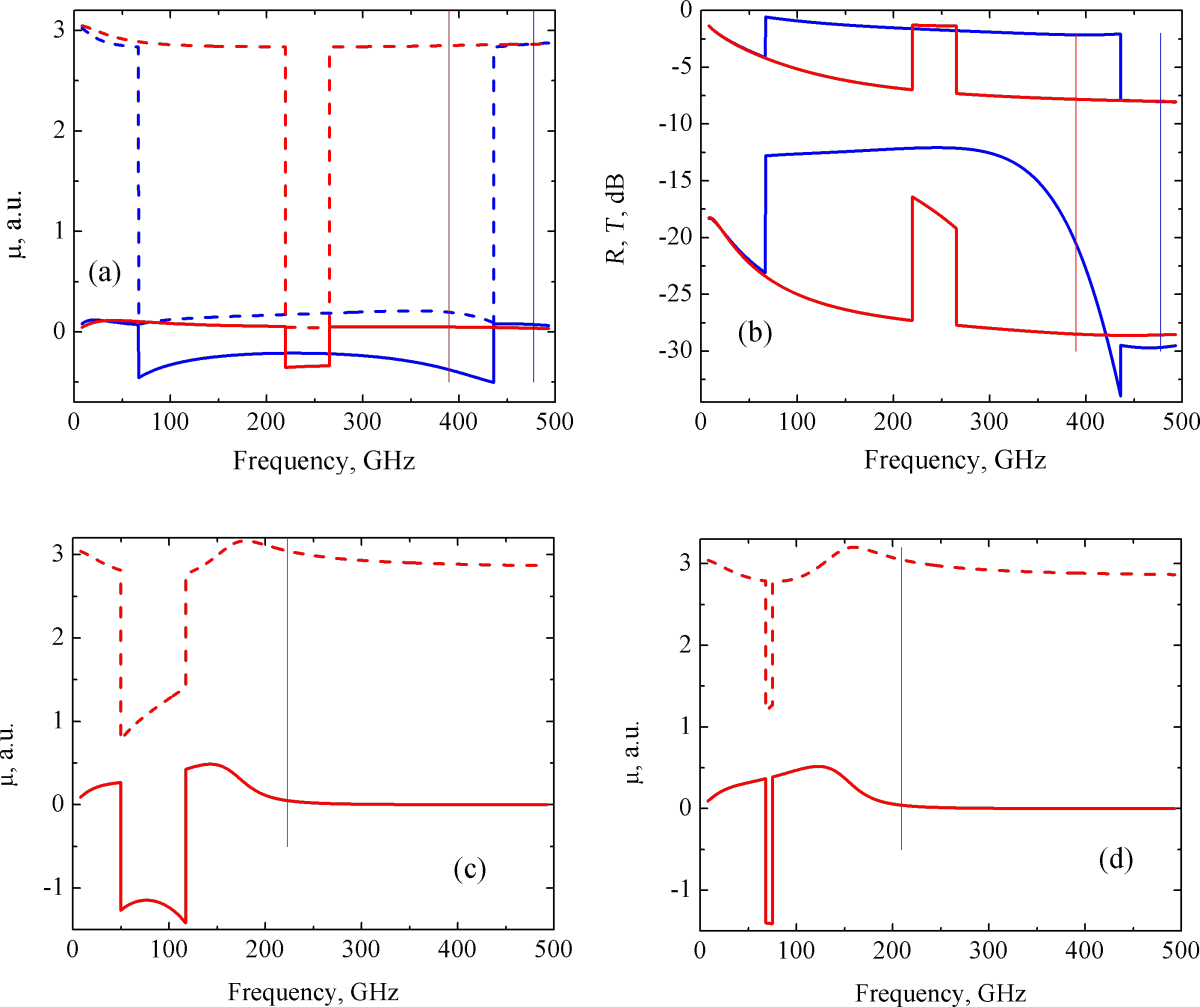
Frequency dependencies of the real (dashed lines) and imaginary (solid lines) parts of the permeability (a,c,d), and of the reflection *R* (upper curves) and transmission *T* (lower curves) coefficients (b) of the CNT based nanocomposite consisting of partially parallel circuit: (a) – *R_i_* = 0.01 Ω, *L_i_* = 0.01 pH, *C_i_* = 10 pF (ω_0_ = 477.5 GHz) – blue curves; *R_i_* = 0.02 Ω, *L_i_* = 0.01 pH, *C_i_* = 10 pF (ω_0_ = 390.0 GHz) – red curves; (b) – *R_i_* = 0.01 Ω, *L_i_* = 0.01 pH, *C_i_* = 10 pF (ω_0_ = 477.5 GHz) – blue curves; *R_i_* = 0.02 Ω, *L_i_* = 0.01 pH, *C_i_* = 10 pF (ω_0_ = 390.0 GHz) – red curves; (c) – *R_i_* = 0.01 Ω, *L_i_* = 0.05 pH, *C_i_* = 10 pF (ω_0_ = 223.0 GHz); (d) – *R_i_* = 0.01 Ω, *L_i_* = 0.0569 pH, *C_i_* = 10 pF (ω_0_ = 209.0 GHz). Resonance frequencies of the *R*_i_*L*_i_*C_i_* circuits ω_0_ are drawn by vertical lines of corresponding colors.

For the partially parallel circuit consisting of a *R_i_* and *L_i_* series connection and *C_i_* in parallel with them, the appearance of distinct steps in the μ, *R* and *T* spectra were obtained for a given set of circuit parameters, as shown in Figure 2. Here, the real part of the permeability reduces to almost zero and its imaginary part becomes negative. The reflection coefficient increases abruptly almost to zero and the transmission coefficient increases from −22 to −10 dB.

Qualitatively, the frequency behavior of the μ, *R* and *T* coefficients is similar to that of the series circuit. However, the width of the valley is much wider in this case and reached the value of 400 GHz. This result is shown in Figure 2a for μ(ω) and in Figure 2b for *R*(ω) and *T*(ω). In subsequent subsections for the other circuit configuration, only μ(ω) curves will be presented.

This wide valley occurs for the following set of given parameters: *R_i_* = 0.01 Ω, *L_i_* = 0.01 pH, and *C_i_* = 10 pF. If we then hold *R_i_* and *C_i_* constant and vary *L_i_* towards higher values, the valley in the μ(ω) dependence is shifted to lower frequencies and narrows, as shown in Figure 2c,d. For values greater than *L_i_* = 0.0569 pH, the valley disappears (not shown here). A similar behavior is obtained when holding *R_i_* and *L_i_* constant and varying *C_i_* in the range 10–41 pF. If instead we set *L_i_* = 0.01 pH and *C_i_* = 10 pF and increase *R_i_* to 0.02 Ω (instead of 0.01 Ω), the valley in the μ(ω) dependence is shifted to the frequency range between 200 and 300 GHz with a corresponding contraction in the middle of the range. This result is depicted in Figure 2a by red line. Note that for the partially parallel circuit, the resonance occurs at frequencies lower than the frequency of the resonance *R_i_L_i_C_i_* circuit.

**Figure 3:**
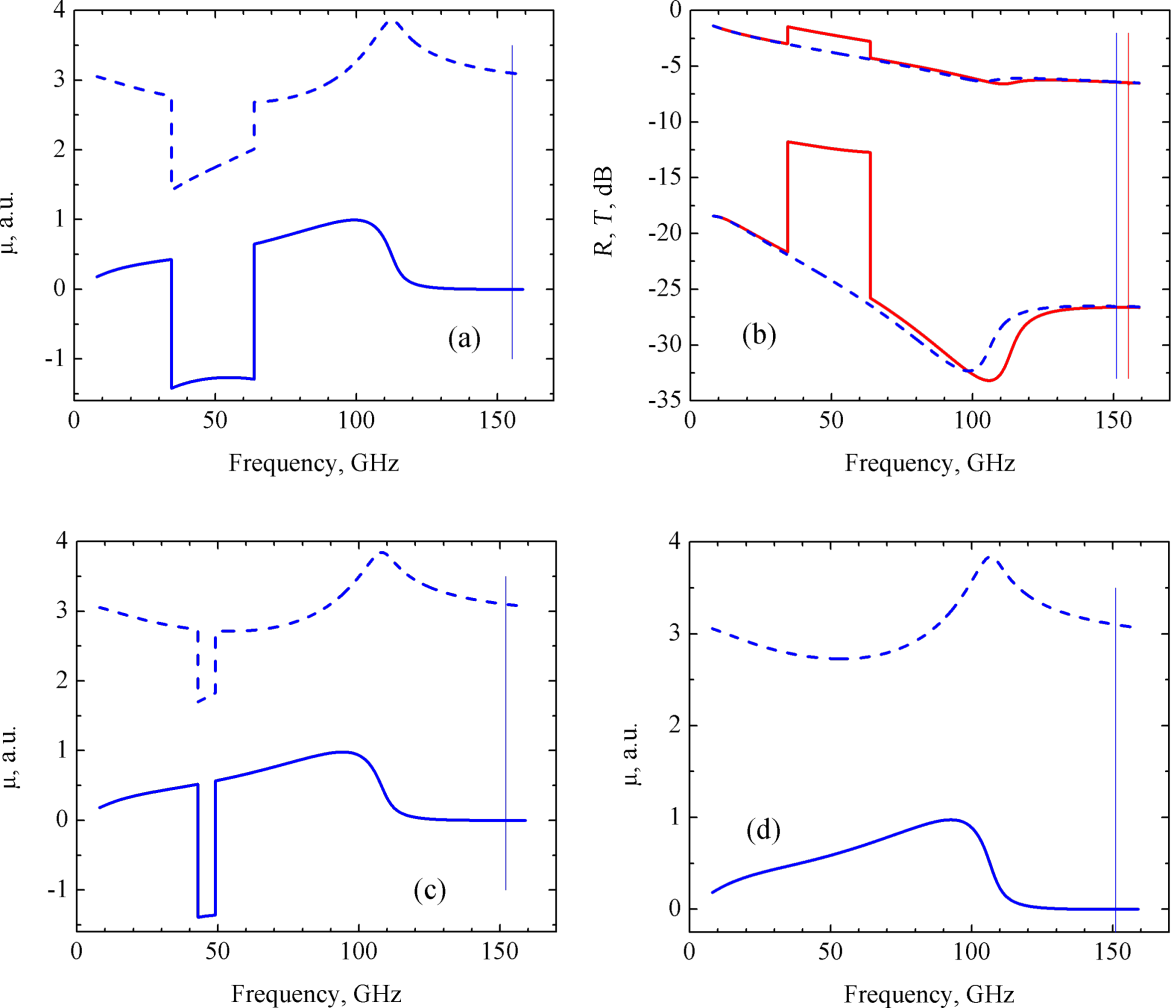
Frequency dependence of the real (dashed lines) and imaginary (solid lines) parts of the permeability (a,c,d), and of the reflection *R* (upper curves) and transmission *T* (lower curves) coefficients (b) of the CNT-based nanocomposite consisting of a series–parallel circuit. (a) *R_i_* = 0.007 Ω, *L_i_* = 0.07 pH, *C_i_* = 15 pF (ω_0_ = 155.3 GHz); (b) *R_i_* = 0.007 Ω, *L_i_* = 0.07 pH, *C_i_* = 15 pF (ω_0_ = 155.3 GHz) (red solid lines) and *R_i_* = 0.007 Ω, *L_i_* = 0.075 pH, *C_i_* = 15 pF (ω_0_ = 151.1 GHz) (blue dashed lines); (c) *R_i_* = 0.007 Ω, *L_i_* = 0.073 pH, *C_i_* = 15 pF (ω_0_ = 152.1 GHz); (d) *R_i_* = 0.007 Ω, *L_i_* = 0.075 pH, *C_i_* = 15 pF (ω_0_ = 151.1 GHz). The resonance frequency of the *R_i_L_i_C_i_* circuits, ω_0_, are indicated by vertical lines.

For the series–parallel circuit, two types of resonances were observed. The first one is related to the valley in the μ(ω) dependence. However, in contrast to the previous cases, the real part of the permeability does not drop to zero, as can be seen in Figure 3a. This valley is related to the increase of both the *R* and *T* coefficients as depicted in Figure 3b by the solid lines. The width of this growth reached a value of 30–40 GHz. The second type of resonance yields a smooth increase of the permeability at higher frequencies (Figure 1a). Therefore, both the abrupt and smooth resonances are observed simultaneously. An increase in any one of the parameters of the *R_i_L_i_C_i_* circuit causes a narrowing of this valley (Figure 3c), and eventually, its disappearance (Figure 3d). In particular, from Figure 3d it follows that for the parameters *R_i_* = 0.007 Ω, *L_i_* = 0.075 pH and *C_i_* = 15 pF, only the smooth resonance remains, which is reflected in the smooth *R*(ω) and *T*(ω) dependence (Figure 3b, dashed lines). Note that in this case, the μ(ω) behavior is very sensitive to the circuit parameters. In particular, the change in the induction from 0.07 pH (Figure 3a) to 0.075 pH (Figure 3d) results in the complete disappearance of the valley in the μ(ω) dependence.

**Figure 4:**
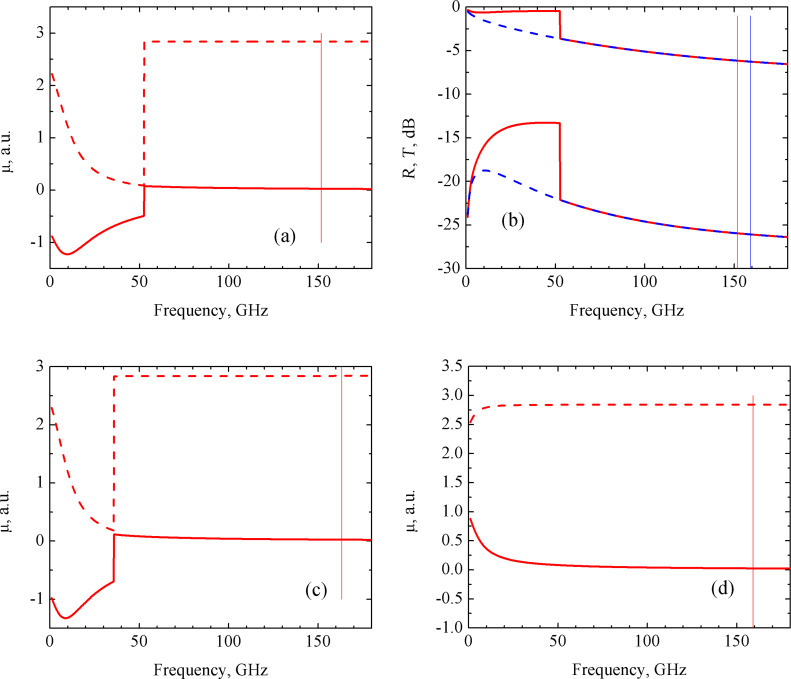
Frequency dependence of the real (dashed lines) and imaginary (solid lines) parts of the permeability (a,c,d), and of the reflection *R* (upper curves) and transmission *T* (lower curves) coefficients (b) of the CNT-based nanocomposite consisting of a fully parallel circuit. (a) *R_i_* = 0.01 Ω, *L_i_* = 0.09 pH, *C_i_* = 10 pF (ω_0_ = 151.7 GHz); (b) *R_i_* = 0.01 Ω, *L_i_* = 0.09 pH, *C_i_* = 10 pF (ω_0_ = 151.7 GHz) (solid red lines) and *R_i_* = 0.01 Ω, *L_i_* = 0.1 pH, *C_i_* = 10 pF (ω_0_ = 159.2 GHz) (dashed blue lines); (c) *R_i_* = 0.01 Ω, *L_i_* = 0.095 pH, *C_i_* = 10 pF (ω_0_ = 163.3 GHz); (d) *R_i_* = 0.01 Ω, *L_i_* = 0.1 pH, *C_i_* = 10 pF (ω_0_ = 159.2 GHz). The resonance frequencies of the *R_i_L_i_C_i_* circuits, ω_0_, are represented by the vertical lines.

In the case of the fully parallel circuit, the steps in the µ(ω), *R*(ω) and *T*(ω) dependence were observed similar to those in the partially parallel circuit of Figure 4. In general, the frequency behavior of the permeability and of the *R* and *T* coefficients differs significantly from that previously observed. In fact, the real part of the permeability first gradually approaches zero, then sharply increases to a value of approximately 3 at ω ≈ 50 GHz, while the imaginary part of μ is negative at ω < 50 GHz and becomes 0 for ω > 50 GHz (Figure 4a). The corresponding behavior of the reflectivity and transmission coefficients is shown in Figure 4b by solid red lines.

It was found that the variation of *R_i_* and *C_i_* within the whole range of interest (see Table 1) does not lead to a noticeable change in the μ(ω) curves. Only a variation of *L_i_* produces a qualitative change in the μ(ω) function. Indeed, when *L_i_* becomes larger than 0.095 pH, the μ(ω) curves become smooth, as shown in Figure 4d. The corresponding frequency behavior of the reflectivity and transmission is depicted in Figure 4b by dashed blue lines.

In our opinion, one of the main results of this work is the observation of intense, wide valleys in the permeability. The reason for the abrupt decrease in the μ(ω) dependence at some combination of the *R_i_L_i_C_i_* circuit parameters is related to the complex contribution to the permeability of the nanocomposite from the circuit and the magnetic NPs. Indeed, the permeability of the nanocomposite is conditioned by 3 main contributions: the superposition of the permeabilities of the carbon matrix, the NPs and the wave impedance of the nanocomposite, as given in Equations 1–3. Here, the permittivity is almost constant. Therefore, the effective nanocomposite parameters are determined both by the carbon matrix parameters and by the polarization of the electrical and magnetic dipoles created by magnetic NPs. These dipoles placed into the carbon matrix cause additional electromagnetic fields which influence the shielding properties of the nanocomposite. Apparently, the determination of the permeability of such nanocomposites should be performed by taking the polarization of such dipoles into account. In our case, the magnetic polarization of dipoles formed by the magnetic NPs is determined by the permeability of the NP and by the impedance of the interface. As a result, during the interaction of the EMR with a composite material having intercalated magnetic metallic NPs of diameter less than the EMR wavelength, additional fields arise in the sample on the length scale much smaller than the EMR wavelength, but larger than the nanoparticle size. These fields are conditioned by the electric and magnetic dipoles created by the metallic NPs. These additional fields should be taken into account when considering the *R* and *T* coefficients. They are accounted for via the effective values of the permittivity and permeability. The values of these fields, or the polarizability, are determined by the nanoparticle–interface–carbon matrix system impedance. At some combination of the resonance circuit parameters and the permeability of the NP, compensation for the absorption occurs and the real part of the permeability goes to zero and its imaginary part becomes negative. In other words, the nanocomposite impedance compensates for the magnetic moment created by the NP in the carbon matrix at a fixed frequency range. When no valley is present in the μ(ω) dependence, the impedance of the magnetic dipole (and consequently, its polarizability) is determined by the permeability of the NP and of the nanocomposite, which are both positive.

## Conclusion

Problems related to the influence of the conductive carbon matrix, the inductive ferromagnetic NPs and the capacitive interface between the carbon matrix and the embedded ferromagnetic nanoparticles in CNT-based nanocomposites on the interaction of these materials with the EMR in the 20–200 GHz frequency range were numerically considered by applying a modified model. A distinctive feature of this model is the introduction of resonant resistive–inductive–capacitive circuits with different connections between the elements to describe the parameters of the nanocomposite. In particular, the circuits were modeled as resonant circuits having resistance, inductance and capacitance with series, parallel and mixed connections.

It was shown that for the series resonance circuit, both smooth and sharp resonances occur in the frequency dependent μ, *R* and *T* coefficients. Depending on the given model parameters, the width of the resonance varied from 1 to 50 GHz. These phenomena occur close to the resonance frequency of the circuit, which is always less than the resonance frequency of the nanocomposite. For the partially parallel circuits, the wide valleys in the frequency dependent μ spectra were obtained. There, the real part of the permeability drops to almost zero, followed by a sharp increase to the value determined by the permeability of the nanocomposite. As a result, over some frequency range, the nanocomposite becomes much more transparent to the EMR. The width of this range reaches hundreds of GHz and it appears at frequencies lower than the resonance frequency of the circuit. For the series–parallel circuits, the nonlinear dependence of μ, *R* and *T* on frequency was obtained. Also, a combination of the nonlinear dependence and the valley-like dependence could be observed for this case depending on the parameters given for the circuits. The valley width reached values of 40 GHz and is extremely sensitive to the model parameter values. They are observed far below the resonance frequency. Finally, for the fully parallel circuit, the μ(ω), *R*(ω) and *T*(ω) dependencies are step-like, but with a slight increase of the resonance frequency of the circuit, these steps disappear and the dependence become gradual.

It is worth mentioning that the obtained effects are in the range of tens and hundreds of GHz due to the characteristic values of the passive elements of the interfaces (pH and pF). Sudden decreases in the μ(ω) coefficient for different *R_i_L_i_C_i_* circuits are explained in terms of the interaction of the magnetic dipole formed by the ferromagnetic metallic NPs and the *R_i_L_i_C_i_* circuits, which leads to the compensation of the resonant absorption of EMR by magnetic dipole.

An experimental observation of the predicted phenomena is possible given improved control of the CNT-based nanocomposite parameters such as size, concentration of NPs embedded into the CNT matrix, NPs localization in the CNT matrix, etc. Further work related to the investigation of the influence of the distribution of the model parameters on the absorption process of EMR by CNT-based magnetic nanocomposite is currently under progress now.
